# Blockade of Catecholamine Reuptake in the Prelimbic Cortex Decreases Top-down Attentional Control in Response to Novel, but Not Familiar Appetitive Distracters, within a Timing Paradigm

**DOI:** 10.3390/neurosci1020010

**Published:** 2020-12-08

**Authors:** Alexander R. Matthews, Mona Buhusi, Catalin V. Buhusi

**Affiliations:** Interdisciplinary Program in Neuroscience, Department of Psychology, USTAR BioInnovations Center, Utah State University, Logan, UT 84322, USA;

**Keywords:** peak-interval, timing, nomifensine, prelimbic cortex, attention, catecholamine

## Abstract

Emotionally charged distracters delay timing behavior. Increasing catecholamine levels within the prelimbic cortex has beneficial effects on timing by decreasing the delay after aversive distracters. We examined whether increasing catecholamine levels within the prelimbic cortex also protects against the deleterious timing delays caused by novel distracters or by familiar appetitive distracters. Rats were trained in a peak-interval procedure and tested in trials with either a novel (unreinforced) distracter, a familiar appetitive (food-reinforced) distracter, or no distracter after being locally infused within the prelimbic cortex with catecholamine reuptake blocker nomifensine. Prelimbic infusion of nomifensine did not alter timing accuracy and precision. However, it increased the delay caused by novel distracters in an inverted-U dose-dependent manner, while being ineffective for appetitive distracters. Together with previous data, these results suggest that catecholaminergic modulation of prelimbic top-down attentional control of interval timing varies with distracter’s valence: prelimbic catecholamines increase attentional control when presented with familiar aversive distracters, have no effect on familiar neutral or familiar appetitive distracters, and decrease it when presented with novel distracters. These findings detail complex interactions between catecholaminergic modulation of attention to timing and nontemporal properties of stimuli, which should be considered when developing therapeutic methods for attentional or affective disorders.

## 1. Introduction

Time estimation is critical for human and animal survival. For instance, the anticipation of an approaching car allows a pedestrian or an animal to know when it is safe to cross. Interval timing—perception of time within the seconds-to-minutes range [1]—is thought to underlie adaptive behaviors such as action, decision making, and rate calculation [2–4]. Diverting attention away distorts timing and impairs cognitive processing and timed responses [5–7].

In interval timing tasks, such as the peak interval procedure, subjects are trained to respond to a particular time criterion when reward is expected. In these procedures, timing can be interrupted by task-irrelevant distracters, such as auditory stimuli, which results in a delay in responding [8–10]. The Relative Time-Sharing (RTS) model [6,9,11] proposes that a limited pool of attentional resources is shared by all cognitive processes including timing. When distracters are presented, attentional resources are diverted away from the primary timing task and reallocated towards the processing of the distracter, which results in a delay in timed responses. Depending on the salience of the distracter [8], the subject may ignore the distracter (“run”, no delay), may interrupt timing during the distracter (“stop”, delay equal to the distracter), may restart timing immediately after the distracter (“reset”) [6], or wait much longer after the distracter before restarting timing (“over-reset”) [5,7,12–14].

The medial prefrontal cortex is thought to play an important role in timing [15–19], as well as in other processes such as inhibitory control, working memory, and decision-making [20–22]. The *prelimbic cortex* (PrL) has been identified as a brain region involved in time discrimination [23] and as a mediator of top-down attentional control during a timing task [7,18]. Presentation of familiar aversive distracters, such as stimuli previously paired with aversive consequences, results in large delays in timing (over-reset) [5], which can be in part alleviated by increasing catecholamine levels within PrL [7]. Individuals with depression show impairments both in time perception [24] and attentional processing [25], both dependent on prefrontal cortex (PFC)[2,26,27], but these impairments are less studies and understood.

Timing is sensitive to a variety of pharmacological manipulations including catecholaminergic drugs [7,28–36]. Stimulants like methamphetamine and cocaine inhibit reuptake of catecholamines and have been shown to alter both timing [28] and attention to time [29]. Some catecholamine reuptake inhibitors, such as nomifensine [37], are effective antidepressants. For example, nomifensine has been shown to reduce immobility both in the forced swim test [38,39] and in the tail suspension test [40] and was marketed as antidepressant in the 1970s and 1980s in the United States under the names Merital/Alival. Although the effects of some antidepressants have been examined in timing paradigms [7,41–43], further elucidation in regard to their effects on attention to time, particularly in paradigms involving distracters, is warranted.

Here we sought to investigate the effects of blockade of catecholamine reuptake by nomifensine within the PrL on the delaying effects of auditory stimuli previously paired with appetitive reinforcers, or presented for the first time during the testing phase, using procedures similar to [14]. Based on previous studies [7], we expected that (a) blockade of catecholamine reuptake would have no effect on timing behavior in the absence of distracters, and that (b) blockade of catecholamine reuptake would enhance top-down attentional control and decrease the timing delays after familiar appetitive or novel distracters, as it does for aversive distracters [7]. The first prediction was confirmed, but the latter was not.

## 2. Materials and Methods

### 2.1. Subjects

The subjects were 26 male Sprague Dawley rats (Harlan Laboratories, Inc., Indianapolis, IN), approximately 3 months old (300–350g) at the start of the experiment. Subjects were randomly assigned to two groups: appetitive and novel and trained in procedures described below. After removing subjects due to misplaced injector tips, poor timing accuracy after surgery, unreliable timing functions, or failure to respond consistently on the nontiming lever, the final number of subjects used in analyses was 17 (appetitive n = 10, novel n = 7). Rats were maintained at approximately 85% of their ad libitum weight by restricting food access in their home cages. Rats were housed in a temperature and humidity-controlled room with a 12/12 light-dark cycle. Water was freely available in the home cage. All experimental procedures were conducted in accordance with the National Institutes of Health’s Guide for the Care and Use of Laboratory Animals and were approved by Utah State University IACUC Committee (protocol 2254).

### 2.2. Apparatus

The apparatus consisted of 16 standard rat operant chambers (Med Associates, St. Albans, VT), housed in sound attenuating cubicles. All chambers were equipped with two levers situated on either side of a pellet receptacle: the left lever was the timing lever for all rats. Rats received 45-mg pellets (Bio-Serv, Flemington, NJ) as rewards. A 28-V 100-mA house light was used as a to-be-timed stimulus. An 80-dB white noise stimulus was used as a distracter stimulus for all rats.

### 2.3. Behavioral Procedures

#### 2.3.1. Interval Timing–Left Lever

Rats were initially reinforced on a *fixed-ratio* (FR)-1 schedule with food pellets for pressing on the timing (left) lever. Rats were then trained on a *reversed fixed-interval* (RFI)-40 schedule [6,45], where the to-be-timed visual stimulus for timing behavior was the absence of the house light. During RFI trials, the first lever response on the timing lever, 40s into the trial, resulted in food delivery and turned on the house light for the duration of the *intertrial interval* (ITI). Afterwards, RFI trials were randomly intermixed with *reversed peak-interval* (RPI) trials, which were unreinforced probe trials during which the to-be-timed stimuli lasted three times the criterion time (120 s) irrespective of responding. Responding on the nontiming (right) lever during this stage of training was inconsequential. Trials were separated by 60 ± 30 s ITIs, during which the house light was illuminated.

#### 2.3.2. Appetitive Noise Training–Right Lever

Rats were randomly assigned to two groups. Group novel was not exposed to the noise until testing and was never rewarded for pressing on the nontiming (right) lever. Group appetitive was (sometimes) reinforced with food pellets for pressing the right lever during the noise, as follows: In separate sessions, appetitive rats were occasionally rewarded for lever pressing during the noise on the nontiming (right) lever in three 3-hr *random-interval* (RI)-16 sessions (16 s noise) with probability 1.0, averaging about 130 trials/session or until rats earned 200 rewards, followed by ten 3-hr RI8 sessions (8 s noise) with probability gradually decreasing from 1.0, to 0.65, and finally to 0.5; the latter RI8 sessions included 150 trials/session or until rats earned 200 rewards. In all, appetitive rats received about 1700 noise presentations prior to testing. One rat was eliminated from the study because it failed to respond consistently on the nontiming (right) lever in the presence of the noise.

### 2.4. Surgery

Rats underwent aseptic surgical procedures under isoflurane anesthesia. Bilateral cannula guides (PlasticsOne, Roanoke, VA) were implanted into the PrL at bregma AP: + 2.50 mm, ML: ± 0.60 mm, DV: −3.50 mm [46] and permanently affixed to the skull. Rats were allowed to recover at least one week before behavioral testing resumed.

### 2.5. Drugs

Catecholamine reuptake blocker *Nomifensine* maleate (NOM) (Sigma Aldrich, St. Louis, MO) was dissolved in vehicle: 45% cyclodextrin (methyl-beta-cyclodextrin) (Sigma Aldrich, St. Louis, MO) in sterile saline. Drugs were prepared fresh prior to all testing procedures.

### 2.6. Local Infusions

Before testing, rats received 0.5 μL infusions of vehicle, NOM 0.4 μg, or NOM 4 μg bilaterally into the PrL. Rats were tested in a Latin-square design to prevent testing biases. Infusions occurred at a rate of 0.25 μL/min for two minutes. Injector cannulae were left in place for two minutes following the infusion.

### 2.7. Testing with and without Noise Distracters

Following drug infusions, rats in both groups were placed in operant boxes and received a 3-hr test session where rats were randomly presented RFI trials, RPI trials, and *reversed peak-interval trials with noise distracter* (RPI + N; for novel rats these were the first trials with a noise presentation). In RPI + N trials, an 8-s noise was presented 8 s into the trial. RPI + N trials were unreinforced, response-independent probe trials lasting 120 s. Appetitive rats received noise retraining sessions in between drug testing sessions.

### 2.8. Histology

**Figure 1.**
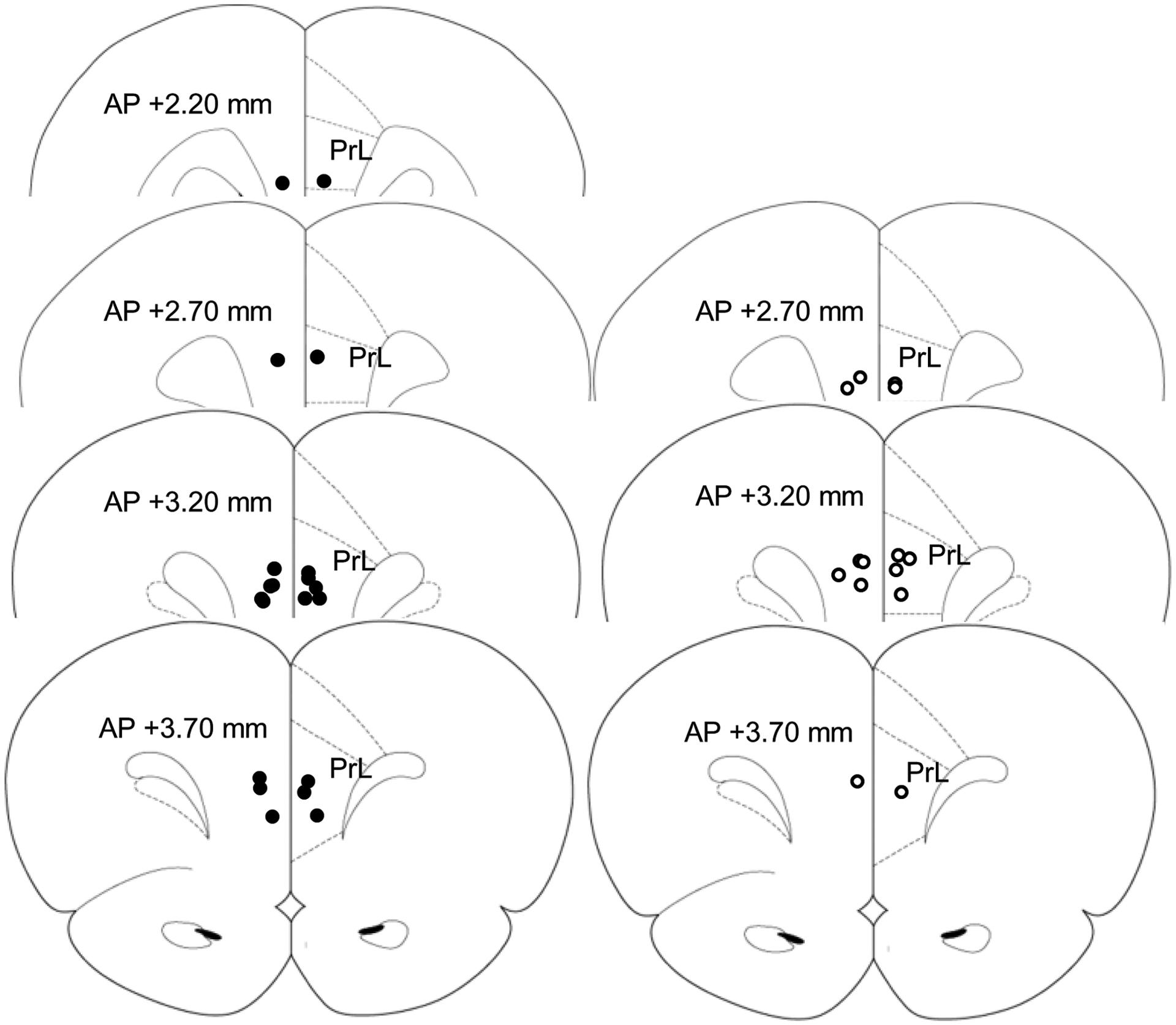
Locations of injector tips targeting the PrL. Brain region outlines are reproduced from [46]. Circles indicate the infusion cannula tip locations. PrL = Prelimbic Cortex. Appetitive = closed circles (left), novel = open circles (right). (appetitive n = 10, novel n = 7).

Rats were transcardially perfused with 10% formalin (Fisher Scientific, Pittsburgh, PA). Brains were sectioned at 60 μm on a vibratome (VT 1200S, Leica). Injector tip placements were identified (see Figure 1). Two rats were removed from the final analyses due to injector tip placements outside the PrL.

### 2.9. Data Collection and Analysis

Lever response data was collected using MED-PC-IV software (Med Associates, St Albans, VT). Timing (left) lever responses in RPI and RPI + N trials in the testing phase were analyzed to determine timing accuracy (estimated peak time, or estimated time of maximal lever responding, thought to be an individual estimate of the criterion duration) [47], and timing precision (estimated width of the response function), using two methods. First, using a *Curve Fitting* (CF) method, individual response curves were fit to a Gaussian [45,48], using the Marquardt–Levenberg algorithm [49] over an 80-s window of analysis: 0–80 s in RPI and 16–96 s in RPI + N trials. The parameters of curve fitting algorithm directly provided estimates for timing accuracy (peak time) and precision (width of the timing function). Second, using the *Interauartile* (IQR) method, responses were divided into 4 quartiles; the average of the 25% and 75% quartiles was taken as an estimate of timing accuracy (peak time), and the time difference between the 25% and 75% quartile was taken as an estimate of timing precision (width of the response function) [6]. We opted for these two methods because they are very different in complexity and assumptions: The CF method assumes a Gaussian-shaped response function, is relatively complex, and it is not intuitive. In contrast, the IQR method is intuitive, easy to understand, and has no assumptions relative to the shape of the response function. Despite being very different in assumptions and complexity, when applied to previous data sets, these methods provided roughly similar estimates for timing accuracy (peak time) and precision (width of the response function) [6,50]. Using each of the two methods, an individual time delay in RPI + N trials relative to RPI trials was calculated as the difference between the estimated peak times in RPI + N and RPI trials. Five rats were removed for poor timing accuracy after surgery (estimated peak time more than 20% off from the criterion time). One rat had very noisy timing functions (coefficient of determination *r*^2^ = 0.42) and was removed from further analyses (see Figure 1; final counts: appetitive n = 10, novel n = 7).

The number of nontiming (right) lever responses, the number of rewards earned in the noise conditioning sessions, and the number of nontiming lever responses during the RPI and RPI + N sessions were collected and analyzed. A response elevation ratio for the right (nontiming) lever was calculated as the ratio between the number of right lever presses made during the 8 s noise presentation period during RPI + N trials and the sum of the responses during the 8-s noise and during the 8-s interval preceding the noise.

### 2.10. Statistical Analyses

Estimated measures of peak time, time delay, response function widths, coefficients of determination (*r*^2^), timing lever, and nontiming lever response rates, number of responses on the nontiming lever during noise presentations, number of rewards earned during noise conditioning sessions, and elevation ratios for the nontiming lever, were analyzed using mixed ANOVAs with NOM dose (0, 0.4, 4 μg) as the repeated measure and group (appetitive, novel) as the between-subjects variable, followed by planned comparisons and Fisher LSD post-hoc analyses. The *alpha* level for analyses was set at *p* = 0.05. Analyses were conducted using Statistica (StatSoft, Inc., Tulsa, OK).

## 3. Results

### 3.1. Appetitive Rats Conditioned to the Noise Stimuli

**Figure 2.**
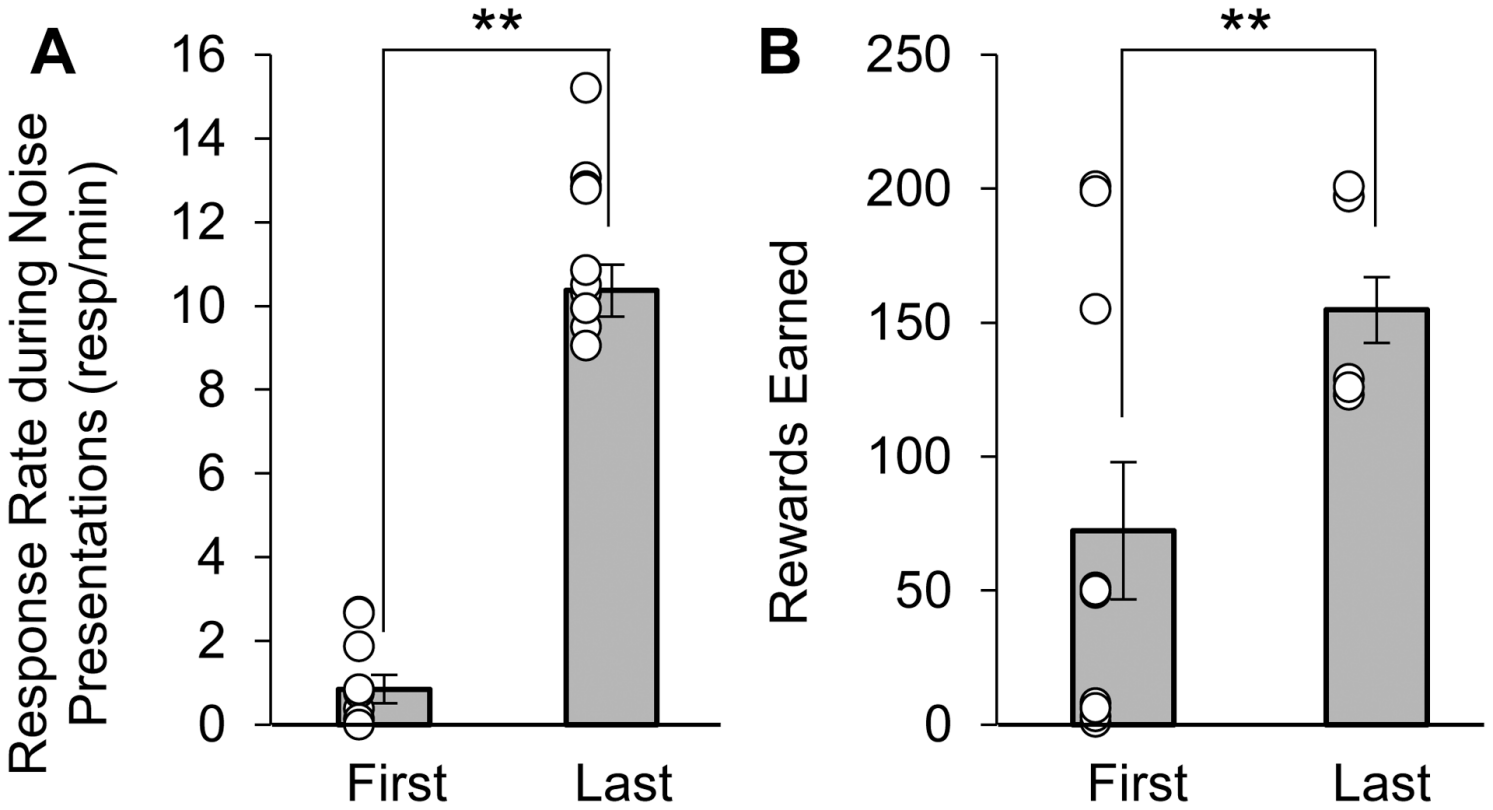
Average response rate (± SEM) and average rewards earned (± SEM) during appetitive noise conditioning sessions. As conditioning progressed from first to last session, the average response rate on the right lever (**A**) and the number of rewards earned (**B**) significantly increased. Open circles indicate individual data points. ** *p* < 0.01.

To confirm that appetitive rats conditioned to the noise stimulus, the response rates and number of rewards received during the appetitive noise training conditioning were analyzed (Figure 2). As the appetitive noise conditioning progressed, the response rate on the nontiming (right) lever during the noise significantly increased from 0.8 ± 0.3 resp/min in the first conditioning session to 10.4 ± 0.6 resp/min in the last conditioning session (Figure 2A) (*F*(1,9) = 246.70, *p* < 0.0001). Additionally, the number of rewards earned significantly increased from 72.3 ± 25.7 in the first conditioning session to 154.8 ± 12.3 in the last conditioning session (Figure 2B) (*F*(1,9) = 16.28, *p* = 0.0030). Taken together, these results indicate that appetitive rats acquired the secondary task (occasionally earning rewards by pressing on the right lever during the noise).

### 3.2. PrL Blockade of Catecholamine Reuptake Did Not Alter Timing Accuracy in Trials without Distracters

**Figure 3.**
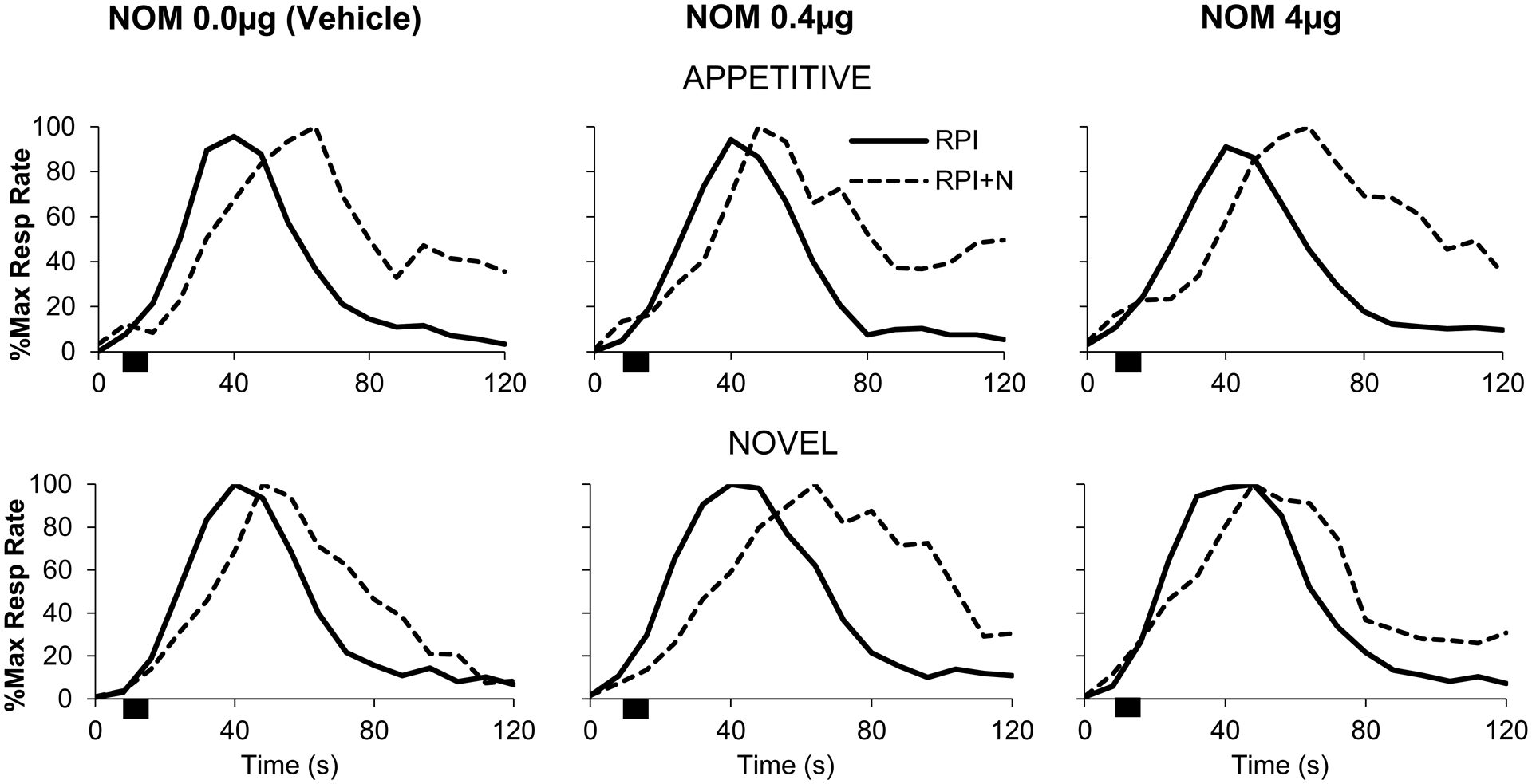
Timing response functions in the appetitive and novel groups under nomifensine (NOM). Timing in RPI (solid lines) and RPI+N trials (dotted lines) in the appetitive (top) and the novel groups (bottom) under the effect of vehicle (left), NOM 0.4 μg (middle), and NOM 4 μg (right). Noise presentations (black rectangles) resulted in timing functions being delayed (shifted rightwards) to a later time.

Figure 3 (solid lines) shows normalized left lever timing functions in RPI trials (without distracters). Irrespective of group or drug dose the response functions in trials without distracters (RPI, solid curves) peaked about the criterion interval (40 s) suggesting that rats acquired the timing task and were not affected by the drug regimen. These suggestions were supported by both analyses using the *Curve Fitting* (CF) method and the *Interquartile Range* (IQR) method.

The CF method provided the following statistics for trials without distracters (RPI, solid curves in Figure 3): Under vehicle, timing functions peaked at 40.1 ± 0.8 s for appetitive rats and at 41.5 ± 1.0 s for novel rats, indicating that rats acquired the timing task. Appetitive rats peaked at 42.0 ± 1.8 s under the NOM 0.4μg, and at 41.2 ± 1.8 s under NOM 4 μg, while novel rats peaked at 41.2 ± 1.2 s under NOM 0.4 μg, and 41.6 ± 0.6 s under NOM 4 μg. A mixed ANOVA of estimated peak times in RPI trials failed to reveal any significant differences between groups, drug conditions, or group x drug interaction, all *F*s < 0.32, all *p*s > 0.73. These results indicate that in peak trials without distracters, the drug regimen did not affect timing accuracy irrespective of the group or the dose.

Very similar statistics were provided for RPI trials using the IQR method. Under vehicle, timing functions peaked at 40.2 ± 1.0 s for appetitive rats and at 41.3 ± 1.0 s for novel rats, indicating that rats acquired the timing task. Appetitive rats peaked at 41.5 ± 1.4 s under the NOM 0.4 μg, and at 42.4 ± 1.1 s under NOM 4 μg, while novel rats peaked at 42.1 ± 1.0 s under NOM 0.4 μg, and 42.3 ± 0.6 s under NOM 4 μg. A mixed ANOVA of estimated peak times in RPI trials failed to reveal any significant differences between groups, drug conditions, or group x drug interaction, all *F*s < 1.81, all *p*s > 0.18. Taken together, these results indicate that, according to both the CF and IQR methods, in RPI trials rats peaked about the criterion interval irrespective of the group or drug dose.

### 3.3. PrL Blockade of Catecholamine Reuptake Significantly Modulated Responding after Novel Distracters in an Inverted-U, Dose-Dependent Manner but Not after Familiar Appetitive Distracters

In RPI + N trials (Figure 3, dotted lines), the 8-s noise distracter was presented 8 s into the trial (indicated by the black rectangle), resulting in delayed timing behavior irrespective of group or drug dose. Figure 3 also indicates that the delay was larger in novel rats at the 0.4 μg NOM dose than in any other group/condition. These suggestions were supported by both analyses using the CF and IQR methods. The time delay in RPI + N trials relative to RPI trials was compared with the behavioral responses of “run” (0-s delay), “stop” (8-s delay), and “reset” (16-s delay).

**Figure 4.**
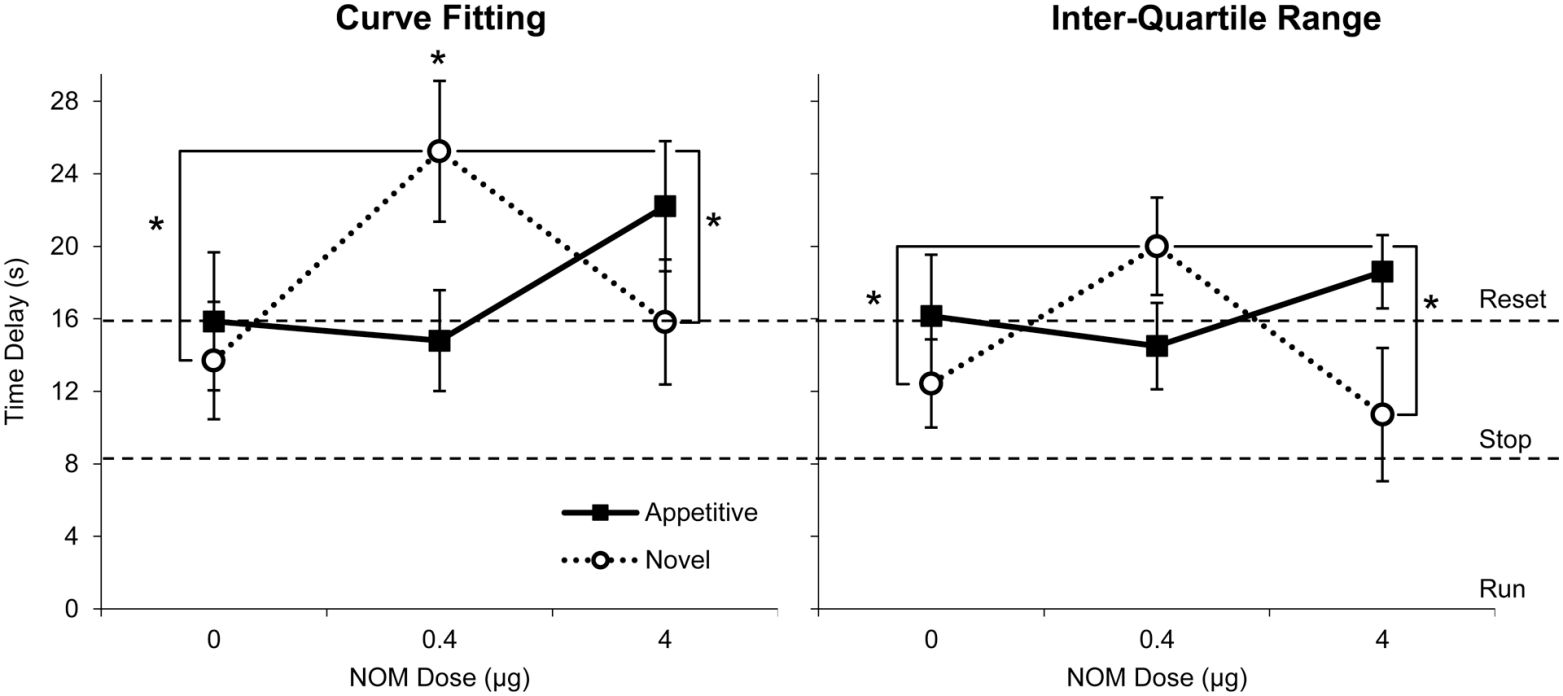
Average time delay (± SEM) under PrL infusion of nomifensine (NOM) estimated by the Curve Fitting method (left) and the Interquartile method (right). Both methods indicated that the time delay in RPI + N trials relative to RPI trials were significantly increased for novel rats (open circles) under the NOM 0.4 μg condition compared to the vehicle and NOM 0.4 μg conditions. Appetitive rats (closed squares) reliably reset under all drug conditions. Dashed lines indicate behavioral responses “run” (0-s delay), “stop” (8-s delay), and “reset” (16-s delay). * *p* < 0.05.

The left panel of Figure 4 shows the estimated delay using the CF method. Appetitive rats delayed 15.9 ± 3.8 s under vehicle, 14.8 ± 2.8 s under NOM 0.4 μg, and 22.2 ± 3.6 s under NOM 4 μg (Figure 4, left panel, closed squares), not significantly different from resetting: Vehicle, *t*(9) = 0.037, *p* > 0.9715, NOM 0.4 μg, *t*(9) = 0.433, *p* > 0.6749, and NOM 4 μg, *t*(9) = 1.735, *p* > 0.1168. Novel rats delayed 13.7 ± 3.2 s under vehicle, 25.2 ± 3.9 s under NOM 0.4 μg, and 15.8 ± 3.5 s under NOM 4 μg (Figure 4, left panel, open circles). Novel rats reset timing under the vehicle, *t*(6) = 0.710, *p* > 0.5043, and under NOM 4 μg conditions, *t*(6) = 0.053, *p* > 0.9597, however, they trended towards over-resetting timing under NOM 0.4 μg, *t*(6) = 2.380, *p* = 0.0548. A mixed ANOVA of time delay indicated a significant drug x group effect, *F*(2,30) = 5.396, *p* = 0.0010, but it failed to indicate significant effects of group, *F*(1,15) = 0.025, *p* = 0.877, or drug *F*(2,30) = 2.174, *p* = 0.131. A Fisher LSD post-hoc analysis indicated that under NOM 0.4 μg, the novel group delayed significantly more than both under vehicle and under NOM 4 μg, *p*s < 0.05, and significantly more than appetitive rats at the same dose, *p* < 0.05 (Figure 3, left panel). Unlike appetitive rats, whose delay was not affected by the drug, novel rats modulated their delay in an inverted-U dose-dependent manner: they delayed significantly more at the NOM 0.4 μg drug dose than under the vehicle and NOM 4 μg conditions.

Similar conclusions were reached using the IQR analysis method (Figure 4, right panel). Using the IQR method, we estimated that appetitive rats delayed 16.1 ± 3.4 s under vehicle, 14.5 ± 2.4 s under NOM 0.4 μg, and 18.6 ± 2.0 s under NOM 4 μg (Figure 4, right panel, closed squares) not significantly different from resetting: Vehicle, t(9) = 0.044, *p* > 0.9656, NOM 0.4 μg, t(9) = 0.628, *p* > 0.5452, and NOM 4 μg, t(9) = 1.285, *p* > 0.2308. Novel rats delayed 12.4 ± 2.4 s under vehicle, 20.0 ± 2.7 s under NOM 0.4 μg, and 10.7 ± 3.7 s under NOM 4 μg (Figure 4, right panel, open circles), not significant from stopping for vehicle, t(6) = 1.823, *p* > 0.1180 and NOM 4 μg, t(6) = 0.737, *p* = 0.4888, and not significantly different from resetting for NOM 0.4 μg, t(6) = 0.148, *p* > 0.1876. A Fisher LSD post-hoc analysis indicated that while appetitive rats’ delay was not significantly different under all drug doses (*p*s > 0.05), novel rats delayed significantly more at the NOM 0.4 μg dose than both under vehicle and under NOM 4 μg, *p*s < 0.05. In summary, both the CF and the IQR analysis methods indicated that the drug did not affect the delay in appetitive rats but modulated the delay in novel rats in an inverted-U dose-dependent manner: novel rats delayed significantly more at the NOM 0.4 μg dose than both under vehicle and under NOM 4 μg in an inverted-U fashion.

### 3.4. PrL Blockade of Catecholamine Reuptake Did Not Significantly Change the Width of the Response Functions

Timing precision was estimated using both the CF and IQR methods: Using the CF method, timing precision (the estimated width of the response function) is one of the parameters of the Gaussian curve fitting; using the IQR method, timing precision can be estimated as the difference between the 75% quartile and the 25% quartile. Because of the inherent differences between the two methods, the two estimates are expected to differ; however, we expect that both estimates will be similarly affected by the group, drug dose, and trial type variables.

A mixed ANOVA of the estimated width of the response function using the CF method [45] failed to indicate a significant main effect of either group, *F*(1,15) = 0.753, *p* > 0.3992, or drug dose, *F*(2,30) = 0.259, *p* > 0.7733, although analyses indicated a significant main effect of trial type, *F*(1,15) = 5.277, *p* = 0.0364, suggesting that the width was significantly larger in RPI+N trials than in RPI trials; analyses failed to indicate any significant interactions, *F*s < 1.460, *p*s > 0.313. Similar results were found using the IQR method [45]: A mixed ANOVA of the estimated width of the response function failed to indicate a significant main effect of either group, *F*(1,15) = 0.700, *p* > 0.4158, or drug dose, *F*(2,30) = 1.256, *p* > 0.2993, although analyses indicated a significant main effect of trial type, *F*(1,15) = 9.783, *p* = 0.0069, suggesting that the width was significantly larger in RPI + N trials than in RPI trials; analyses failed to indicate any significant interactions, *F*s < 1.509, *p*s > 0.2374. Taken together, although timing precision was significantly lower (width of the timing function was significantly larger) in RPI + N trials relative to RPI trials, both methods indicated that timing precision was not affected by neither drug nor group. Thus, the differences in timing delay under the drug between groups cannot be explained by differences in timing precision.

### 3.5. PrL Blockade of Catecholamine Reuptake Did Not Significantly Change the Coefficients of Determination (r^2^) of the Response Functions

Using the CF method, one estimates timing accuracy (peak time) and timing precision (width of the response function) by fitting a Gaussian curve onto the individual response curve in each condition. The goodness of fit is estimated by the coefficient of determination (*r*^2^). A high coefficient of determination indicates a good fit with a Gaussian curve, while a low coefficient of determination indicates that the individual rat’s response curve is noisy, and it is poorly fit by a Gaussian. As detailed in Methods, one rat had very noisy timing functions (*r*^2^ = 0.42) and was removed from analyses.

The *r*^2^ values for the appetitive group were 0.97 ± 0.01 in RPI trials and 0.85 ± 0.04 in RPI + N trials under vehicle, 0.96 ± 0.02 in RPI trials and 0.82 ± 0.04 in RPI + N trials under NOM 0.4 μg, and 0.94 ± 0.02 in RPI trials and 0.84 ± 0.03 in RPI + N trials under NOM 4 μg. Goodness of fit values for the novel group were 0.98 ± 0.01 in RPI trials and 0.86 ± 0.03 in RPI + N trials under vehicle, 0.93 ± 0.01 in RPI trials and 0.70 ± 0.08 in RPI + N trials under NOM 0.4 μg, and 0.94 ± 0.02 in RPI trials and 0.88 ± 0.04 in RPI + N trials under NOM 4 μg. A mixed ANOVA analysis of the *r*^2^ values failed to indicate a significant main effect of either group, *F*(1,15) = 0.437, *p* > 0.5186, drug dose, *F*(2,30) = 2.831, *p* = 0.0748, or any interactions, all *F*s < 2.857, *p*s > 0.0732. There was however a significant main effect of trial type, *F*(1,15) = 44.068, *p* < 0.0001, suggesting rats’ response functions in RPI + N trials were significantly noisier (*r*^2^ = 0.84 ± 0.02) than in RPI trials (*r*^2^ = 0.95 ± 0.01). In summary, although the goodness of fit (coefficient of determination, *r*^2^) was slightly, but significantly, lower in RPI + N trials relative to RPI trials, it was not affected by neither drug dose nor group. Therefore, the differences in timing delay under the drug between groups cannot be explained by differences in goodness of fit.

### 3.6. PrL Blockade of Catecholamine Reuptake Did Not Affect Lever Pressing on either the Timing or Nontiming Levers

#### 3.6.1. Timing Lever

Mixed ANOVAs of timing (left) lever response rates in RPI and RPI+N trials failed to indicate drug, group or drug-group interactions within either RPI (all *F*s < 0.21, *p*s > 0.70) or RPI+N trials (all *F*s < 2.33, *p*s > 0.11). Analyses also indicated a significant effect of trial type on response rate, *F*(1,15) = 66.97, *p* < 0.0001, but they failed to indicate any trial x group effects, trial x drug effects, or drug x trial x group effects, all *F*s < 1.19, *p*s > 0.32, indicating that noise presentations significantly decreased the response rate on the timing (left) lever in RPI+N trials relative to RPI trials under all drug or group conditions. However, the rate of responding on the timing lever was independent of drug manipulation.

#### 3.6.2. Nontiming Lever

**Figure 5.**
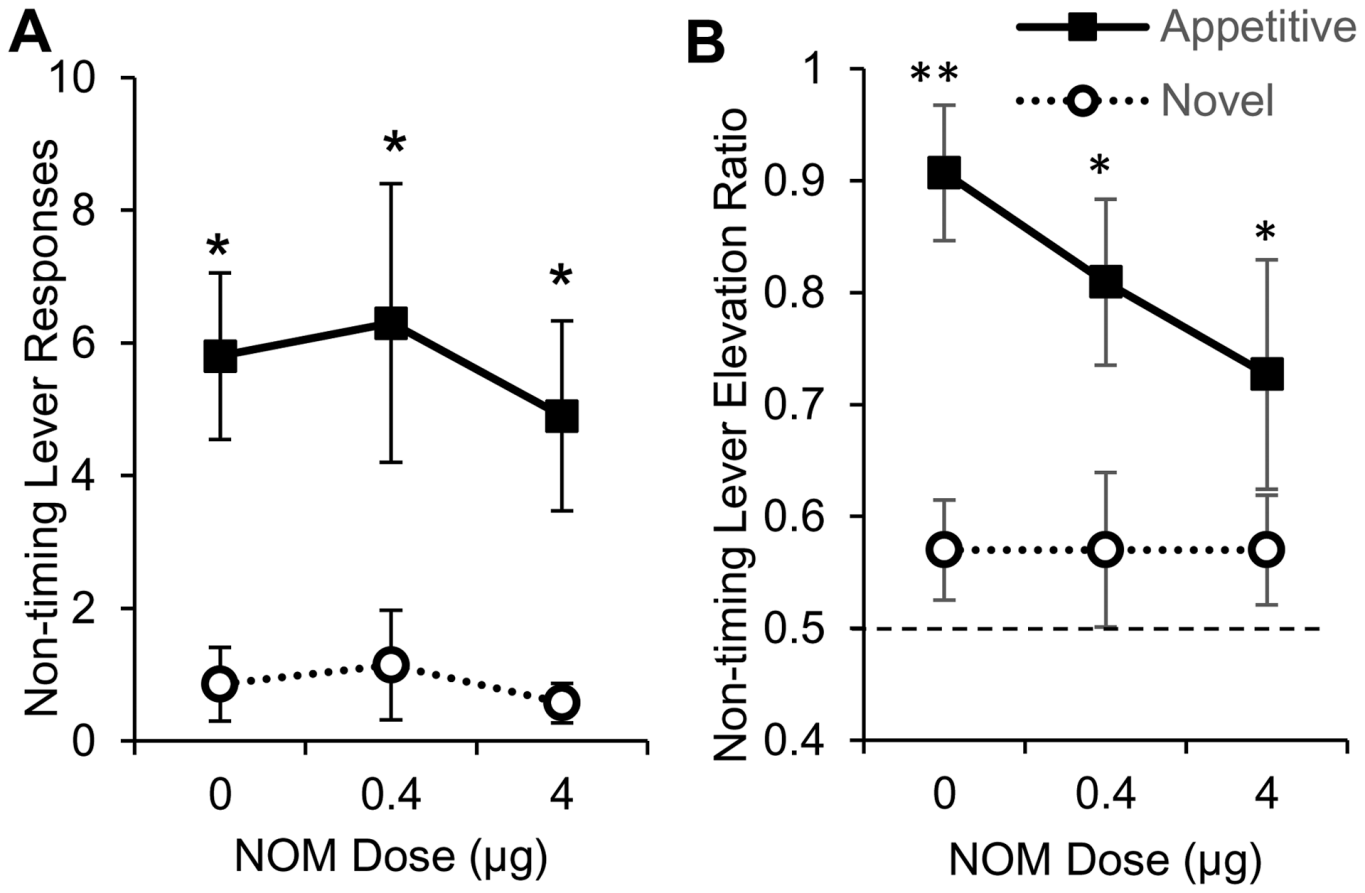
Nontiming lever pressing in RPI+N trials was not affected by NOM. (**A**) Average number of responses (± SEM) on nontiming (right) lever during RPI + N trials in appetitive (closed squares) and novel rats (open circles). (**B**) Response elevation ratio (± SEM) in appetitive (closed squares) and novel rats (open circles). Appetitive rats made significantly more responses on the nontiming lever during the noise than novel rats (A) and increased their responding during the noise relative to prenoise levels (B). Novel rats did not lever press reliably during the noise (A) and responded at chance levels (ratio 0.5, dashed line) during the noise relative to prenoise levels (B). The rate of responding was not affected by NOM dose in either group. ** *p* < 0.01, * *p* < 0.05.

A mixed ANOVA of the number of responses on the nontiming (right) lever during RPI trials (without noise distracter) failed to indicate significant effects of group, drug, or interactions, all *F*s < 2.52, *p* > 0.096, suggesting that, in trials where the noise was not presented, both appetitive and novel rats responded similarly (at very low levels) on the nontiming lever under all drug conditions. However, in trials with noise presentations (RPI + N), analyses of nontiming lever responding indicated a significant effect of group, *F*(1,15) = 11.67, *p* = 0.0038, with no reliable drug or drug x group interaction effects, all *F*s > 0.30, *p* > 0.74, suggesting that appetitive rats responded significantly more than novel rats irrespective of drug dose and that the level of responding (high in appetitive and low in novel) was not affected by the drug dose (Figure 5A).

To further investigate possible drug-related changes in the rate of nontiming lever responding in RPI + N trials, a response elevation ratio was computed by dividing the number of responses during the 8-s noise by the number of right lever responses during the 8-s noise and the preceding 8-s interval (Figure 5B). Figure 5B shows that appetitive, but not novel rats, significantly elevated their nontiming lever rate of response compared to chance level (elevation ratio 0.5). A mixed ANOVA of the elevation ratio indicated a significant main effect of group, *F*(1,15) = 14.798, *p* = 0.0016 but no effect of drug or drug x group interaction (all *F*s < 0.33, *p* > 0.71). Planned comparisons between the appetitive and novel groups’ elevation ratios indicated significant differences under vehicle, *F*(1,15) = 11.246, *p* = 0.0044, under NOM 0.4 μg, *F*(1,15) = 5.340, *p* = 0.0355, and under NOM 4 μg, *F*(1,15) = 4.856, *p* = 0.0436. Overall, appetitive rats significantly increased responding on the nontiming lever relative to prenoise levels when the noise was presented during RPI + N trials, while novel rats responded on the nontiming lever at low levels both before and during the noise. Most importantly, despite group and trials differences, the response rates on either the timing (left) lever or the nontiming (right) lever were not significantly affected by PrL catecholamine manipulations. Therefore, the differential effect of the drug on time delay in novel rats at the NOM 0.4 μg dose (Figure 4) cannot be simply attributed to changes in the responding on either lever in either group, to changes in timing precision, or to changes in goodness of fit, but is more compatible with a change in top-down attentional control to timing vs. processing the distracter.

## 4. Discussion

We adapted a peak-interval timing paradigm with distracters to explore the effects of local catecholamine reuptake blockade in the PrL on timing accuracy and on the top-down attentional control of timing when presented with familiar appetitively-conditioned or novel noise distracters. Based on a previous study which investigated the effect of PrL catecholamine reuptake blockade on timing with aversive distracters [7], we expected that (a) PrL infusion of catecholamine reuptake blocker nomifensine (NOM) would not affect timing accuracy (in trials without distracters), and that (b) PrL infusion of NOM would *reduce* the delaying effect of appetitive or novel noise distracters on timing (in trials with distracters). While the first prediction was confirmed, the latter was not.

Overall, our study revealed a double-dissociation of the effect of PrL catecholamine reuptake blockade on timing between trials with or without distracters and between familiar appetitively-conditioned distracters (appetitive group) or novel distracters (novel group). In trials without distracters, PrL infusion of NOM had no effect on timing accuracy, timing precision, or response rate on either lever in either group. Although NOM has been shown to increase *dopamine* (DA) accumulation in the frontal cortices, brain areas related to timing [23,51,52], and although DAergic drugs alter timing [2], we found no effect of NOM on timing accuracy and timing precision in trials without distracters in either novel or appetitive rats. These findings can be explained by the fact that although nomifensine acts as an indirect DA agonist, binding and blocking DAT and NET similar to amphetamine, it does not have amphetamine’s stimulatory effects on DA release [53], as NOM does not reverse the DA pump direction [54,55]. Therefore, while amphetamine [28,33,56] affects timing accuracy, NOM failed to show such an effect in both the present study and in previous studies involving either rats [7] or healthy human participants [57].

Instead, in trials with distracters, not only did PrL infusion of NOM *fail to reduce* the timing delay after these distracters as predicted by [7] but also significantly *increased* the timing delay after novel distracters in a dose dependent manner, while *failing to alter* the time delay following familiar appetitive distracters (Figure 4). Indeed, appetitive rats reset (restarted) their timing immediately following the noise irrespective of NOM dose; instead, under NOM novel rats delayed their timing in an inverted-U manner: They reset (restarted) their timing following the novel noise at 0 and 4 μg NOM dose but significantly delayed their responding at the 0.4 μg NOM dose (Figure 4). These results cannot be explained by changes in lever pressing on the nontiming lever, since NOM failed to alter responding on this lever irrespective of dose (Figure 5A,B). Moreover, these results cannot be explained by changes in timing precision (width of response function) or in goodness of fit (coefficient of determination, *r*^2^). Taken together, these results suggest that during distracter presentations PrL blockade of catecholamine reuptake did not affect top-down attentional control in appetitive rats, but decreased top-down attentional control of timing in novel rats in an inverted-U fashion, without having any effects on the general timing ability of the subject.

The nonlinear inverted-U dose-response curve is a signature of activation of frontal cortex in tasks requiring attentional control or working memory in humans [58], monkeys [59], and rodents [60]. NOM blocks catecholamine reuptake and indirectly increases activation of both DA and *norepinephrine* (NE) receptors. Therefore, NOM’s inverted-U dose-response curve in novel rats may be due to differential activation of receptors on one or both neurotransmitter systems (e.g., differential affinity towards DAT and NET [61]), activation of nonlinear signaling cascades (e.g., D1 receptors and their cAMP intracellular signaling [59]), regulation of transporter function and surface expression [62], or activation of autoreceptors and reduced neurotransmitter release (NOM has affinity towards alpha-2 adrenergic receptors [63–66]). Our study cannot differentiate between these possibilities, but it provides yet another example in which prefrontal cortex DA and/or NE show a nonlinear cognitive control, generating a dynamic balance between focused attention and flexible attention [67]: Too little or too much PFC activation may have appetitive and novel rats “reset” (restart) timing immediately after the distracter in an “automatic” manner, without processing of the distracter. Instead, at an intermediate “optimal” PFC activation level provided by NOM administration, novel rats may “flexibly” process the distracter and fail to *immediately* return their attentional resources to timing, thus further delaying timing beyond a reset.

Alternatively, novel rats may have experienced the novel noise distracter as an *orienting stimulus* [68], which may have prompted them to investigate the novel stimulus, its location, and/or its possible consequences or significance. The Relative Time-Sharing (RTS) model [6,9,11] proposes that a limited pool of attentional resources is shared by all cognitive processes including timing. When distracters are presented, attentional resources are diverted away from the primary timing task and are reallocated towards the processing of the distracter [9,11]. In novel rats, PrL blockade of catecholamine reuptake may have exacerbated the attentional resources allocated to the novel distracter (orienting stimulus), its location, or its possible consequences even after the distracter [69], such that attentional resources were reallocated back to timing with a significant delay after the distracter, which resulted in time delays beyond a reset (Figure 4). Because in our study NOM was only effective in novel rats, it suggests that PrL catecholamine blockade may have affected exploration following novel stimuli [69] or may have resulted in novel rats being apprehensive (anxious) of the novel noise [70]. Indeed, NOM administration has been previously shown to result in anxiety-like behaviors, such as an increase in novelty-induced hypophagia when food is presented in the middle of a novel open field area [71].

When contrasting the results of the present study with those reported in a previous study in our lab [7], it may be puzzling to note that (a) in the present study rats delayed timing after the neutral distracter while they failed to do so in [7] and (b) in the present study NOM further delayed timing after the neutral distracter while it failed to do so in [7]. Both discrepancies are easily accounted for by the fact that in the present study rats were not presented with the neutral distracter before the test, and as such during the test the neutral distracter was a novel stimulus of unknown consequence, a stimulus that attracts attention [72]. Such a stimulus is expected to delay timing because its processing requires attentional resources which are taken away from processing time [9], as well as be affected by NOM because it engages attentional control circuits in PFC responsible for resource allocation. In contrast, in [7] rats were exposed to the neutral distracter before the test (pre-exposed); by being pre-exposed, the neutral distracter became a stimulus-of-no-consequence, to-be-ignored (latent inhibition [73]). The presentation of such a stimulus is neither expected to affect behavior (e.g., delay timing) nor to be affected by NOM because it does not engage attentional control circuits in PFC.

It is interesting to note that in the present study, the “novel” distracter was repeatedly presented during testing, yet its time-delaying properties failed to diminish. This may be due to its small number of presentations during testing, as well to the relatively unknown relationship between “novelty” and “time-delaying properties”. While there is strong evidence that the time delay produced by an interrupting event increases with discriminability (an element of “novelty”) [74–77], only one experimental investigation of the relationship between time delay and the number of presentations of the event exists to date. To some surprise, we found that under specific conditions distracters may retain their time-delaying properties despite considerable numbers of presentations of the distracter [50], a finding that parallels the fact that under specific experimental conditions repetition *enhances* rather than *diminishes* the “novelty” of a stimulus (reviewed in [78]). The latter finding is supported by computer simulations showing that “novelty” does not simply decrease with repetitions but that it may *increase* rather than *decrease* with repeated presentation of a stimulus (or under specific physiological conditions) [79]. Taken together, discrepancies between this study and previous studies are likely to be accounted for by details of the procedure, possibly by the neutral stimulus in this study being “novel” and engaging attentional processing, while in [7] the neutral stimulus was “pre-exposed”, thus failing to engage attentional processing and delay timing.

In summary, the selective impairment in top-down attentional control of timing after novel, but not appetitive distracters, suggests that PrL catecholamine blockade does not affect timing by altering timing or reward processes but rather processes related to novelty processing [80], increased exploration following novel stimuli [69], or possible defensive or anxiety-related processes activated by novel, unexpected stimuli [81].

## 5. Conclusions

Here we document that prelimbic catecholaminergic top-down attentional control of timing depends upon the valence of distracters. In this study, PrL blockade of catecholamine reuptake by NOM neither altered timing accuracy in trials without distracters nor affected attentional control in rats presented with an appetitive distracter. However, contrary to its beneficial effects on attentional control of timing when presented with aversive distracters [7], PrL blockade of catecholamine reuptake impaired top-down attentional control of timing when presented with novel distracters in an inverted-U, dose-dependent manner. It stands to reason that the valence of attentional distracters, drug dosage, and the specific medications used to alleviate their effects must be carefully considered when prescribing medications to patients with attentional or affective disorders. Future research must be performed to clarify valence-related aspects of attentional disorders and the medications commonly prescribed to lessen their effects.
